# Quality assessment of automatically planned O‐Ring linac SBRT plans for pelvic lymph node metastases, finding the optimal minimum target size by comparison with robotic SBRT

**DOI:** 10.1002/acm2.14143

**Published:** 2023-09-22

**Authors:** Katerine Viviana Díaz Hernández, Sergejs Unterkirhers, Uwe Schneider

**Affiliations:** ^1^ Science Faculty University of Zürich Zürich Switzerland; ^2^ Medical Physics Radiotherapy Hirslanden Zürich Switzerland

**Keywords:** CyberKnife, Halcyon, O‐Ring linac, pelvic SBRT, robotic SBRT, VMAT

## Abstract

**Purpose:**

The purpose of this study is to assess the quality of automatic planned O‐Ring Halcyon linac SBRT plans for pelvic lymph node metastases and to establish an absolute PTV volume threshold as a plan quality prediction criterion. Compliance of the plans to institutional SBRT plan evaluation criteria and differences in plan quality and treatment delivery times between Halcyon Linac and CyberKnife robotic SBRT were evaluated.

**Methods:**

Twenty‐one CyberKnife treatment plans were replanned for Halcyon. Prescription doses range was 26–40 Gy in mean three fractions. The mean/median planning target volume was 4.0/3.6 cm^3^. Institutional criteria for the plan evaluation were: New Conformity Index (NCI), Conformity Index (CI), Modified Gradient Index (MGI), selectivity index reciprocal (PIV/TV_PIV_), and the target coverage by prescription isodose (%PIV). Statistical analysis based on the receiver operating characteristic (ROC) curve was used to determine a plan quality predictor threshold of the PTV volume. Comparative analysis of normal tissue complication probabilities (NTCP) was used to assess the risk of toxicity in healthy tissues.

**Results:**

Seventy‐one percent (*n* = 15)/95% (*n* = 20) of Halcyon and 81% (*n* = 17)/100% (*n* = 21) of CK plans fulfilled all ideal/tolerance criteria. For PTVs above a found *optimal* threshold of 2.6 cm^3^ (71%, *n* = 15), no statistically significant difference was observed between the CI, NCI, PIV/TV_PIV_, and MGI indexes of both groups, while the coverage (%PIV) was statistically but not clinically significantly different between cohorts. Significantly shorter delivery times are expected with Halcyon. No significant differences in NTCP were observed.

**Conclusion:**

All but one automatically optimized Halcyon treatment plans demonstrated ideal or acceptable performance. PTV threshold of 2.6 cm^3^ can be used as decision criteria in clinical settings. The results of our study demonstrated the promising performance of the Halcyon for pelvic SBRT, although plan‐specific QA is required to verify machine performance during plan delivery.

## 1 INTRODUCTION

Stereotactic body radiation therapy (SBRT) is an advanced radiotherapy technique that delivers high doses of ionizing radiation in a limited number of fractions to precisely conform to small and well‐defined targets within the body. This results in a high biologically effective dose within the target and rapidly falling off doses away from the target, potentially maximizing tumor control and minimizing normal tissue damage. SBRT is widely used for the treatment of a wide variety of other primary and metastatic cancers.^1^, ^2^, ^3^, ^4^ In particular, for pelvic lymph node (LN) oligometastases, one‐year local control of 74%^5^ or higher^6^ can be achieved with a mild risk of toxicities. For the clinical performance of SBRT, dedicated systems and regular clinical linear accelerators (Linacs) are employed.

Recently Varian Medical Systems developed an alternative to traditional C‐arm Linacs, the fast‐rotating O‐ring Linac Halcyon, to improve delivery speed, treatment accuracy, and patient safety.^7^, ^8^ The device features a dual‐layer multileaf collimator (MLC) SX2 with a maximum leaf speed of 5 cm/s, a double stack configuration that reduces MLC leakage and decreases the transmission dose to less than 0.5%. It also equipped with a fast kilovoltage cone‐beam computed tomography (CBCT) imaging, a 6 MV FFF therapeutic radiation beam and has a rotational speed four times higher than C‐Arm Linacs.^9^

Thus, Halcyon has the potential to increase patient throughput, and the performance of SBRT on such high yield machines, which can be very beneficial in busy clinic settings. Furthermore, shorter delivery times offer potential advantages in minimizing intrafraction motion. Existing studies focused on investigating the use of Halcyon Linac for SBRT treatments of targets of lung, spine, abdominal, and pelvic localizations.^8^, ^10^, ^11^ In particular, Halcyon has already been used to treat pelvic oligometastatic LN lesions using the SBRT technique, obtaining plan dosimetric results comparable to the Varian TrueBeam Linac with higher treatment delivery speed for targets with mean/median 18.84/19.66 cm^3^ combined volume (range 5.57–39.5 cm^3^).^11^ Treatment of smaller pelvic volumes has not been investigated so far.

In contrast, the CyberKnife (Accuray Incorporated, Sunnyvale, California, USA) was developed as a dedicated device for the performance of Stereotactic Radiotherapy and Radiosurgery while it can also be used for delivery of conventionally fractionated Radiotherapy.^12^ It consists of a single‐energy flattening filter‐free (FFF) 6 MV Linac mounted on a high‐precision robotic arm combined with an in‐room stereoscopic x‐ray imaging system. The image‐guided six degrees of freedom movement arm enables tumor tracking aided by the bony structures and implanted fiducials. Additionally, this device is capable of markerless tracking of lung tumors that have sufficient x‐ray contrast. This system has been widely used to treat pelvic LN, among other malignancies, with excellent results.^13^, ^14^

This retrospective replanning study investigates the feasibility of Halcyon treatments for pelvic LN using SBRT by comparing 21 replanned Halcyon treatment plans with the original ones that were delivered using the CyberKnife (CK) system and establishing the absolute PTV volume threshold as the plan quality prediction criteria for Halcyon plans, as its performance can be limited with MLC leaf resolution. The clinical CK plans used in this study followed institutional guidelines based on the recommendations of the ICRU report 91^15^ and the Stereotactic Ablative Radiotherapy Consortium (SABR) UK protocol.^16^ The dosimetric performance of both cohorts of treatment plans was evaluated using parameters such as the new conformity index (NCI), the conformity index (CI), the modified gradient index (MGI), the fraction of the prescription isodose volume (PIV) to the target volume within the prescription isodose volume (TV_PIV_), and the percentage PIV. Furthermore, the risk of toxicity to organs at risk (OARs), such as the bladder, rectum, and small bowel, was evaluated by comparing the normal tissue complication probabilities (NTCP) estimated with the Lyman Kutcher Burman (LKB) and relative seriality (RS) models. So far, except and excluding prostate SBRT, where the target is of substantial size,^17^ no studies have been published comparing CK and Halcyon for SBRT treatment. To our knowledge, this is the first investigation comparing these two systems for SBRT treatment of metastases in pelvic LN.

## 2 METHODS AND MATERIALS

### 2.1 Patient sample

In this study, a total of 20 consecutive patients (21 treatment plans) who underwent SBRT treatments on the CK system for pelvic LN metastases in our department between November 2020 and October 2022 were selected. One of the cohort patients was treated twice, constituting 21 LN SBRT plans. The planning target volumes (PTV) were created by adding a margin to the delineated gross tumor volumes (GTV). The OARs considered for plan comparison were the skin, bladder, rectum, and small bowel. Additional structures, when contoured, were not considered for plan evaluation.

### 2.2 Treatment plans

The planning computed tomography scan (CT) was acquired in the supine position. The CT region was from mid‐abdomen to mid femoral bones. The slice thickness was 1.5 mm. For all patients, the SOMATOM Confidence CT scanner (Siemens Healthineers, Erlangen, Germany) was used for CT data acquisition. All patients had implanted fiducials (Gold Anchor, Naslund Medical AB, Huddinge, Sweden) for tracking target during patient positioning and treatment delivery.

Original CK radiotherapy plans for all patients were developed using the Precision treatment planning system (TPS) (Precision versions 3.0 and 3.3; Accuray Inc, Sunnyvale, California, USA). For treatment, fixed collimators robotic SBRT plans were used. Arms were blocked for beam entry for all patients, and plans were calculated using the RayTracing algorithm with high calculation resolution (highest voxel dimension 1.5 mm).

The Halcyon plans were automatically generated through the Eclipse Scripting Application Programming Interface (ESAPI) in the Eclipse treatment planning system (TPS) (Eclipse version 16.1.0, Varian Medical Systems, Palo Alto, California, USA). A template was created using two full arcs with a 6 MV FFF beam (800 MU/ min) to produce volumetric modulated arc therapy (VMAT) plans. Two‐full arcs setup was employed for auto‐planned plans, as was already utilized by Pokhrel et al. for prostate SBRT with Halcyon Linac.^18^ Beforehand the Halcyon couch was inserted as a structure and patients’ arms were removed from the Body structure if present. The isocenter was located at the geometrical center of the PTV in the case of single tumors, and for dual targets, the isocenter was automatically aligned in Eclipse based on the combined tumor volume. The collimator angles were chosen as 30^◦^ and 330^◦^ to reduce the MLC tongue‐and‐groove leakage dose to normal tissue according to the vendor instructions.^19^ Three rings of 4 mm thickness, each separated by 2 mm, were introduced as helper structures to force dose conformity. The photon inverse planning optimizer PO 16.1.0 HAL and the photon dose calculation algorithm AcurosXB 16.1.0 HAL with a calculation resolution of 1.25 × 1.25 × 1.25 mm^3^ were used. The optimization goals were applied to the rings and PTV to achieve a coverage higher than or equal to 95% of the prescription isodose while maintaining sufficient conformity. The template included a second optimization of the PTV in order to increase the coverage. No further optimization of the plans was performed. In addition, the treatment prescription isodose was selected in the range of 66–75% to cover 95% of the target volume whenever possible. All Halcyon SBRT plans followed the institutional dosimetric constraints based on the SABR UK protocol,^16^ Timmerman,^20^ HYTEC^21^ publication, and AAPM report 101,^22^ which are summarized in Table 1. For two‐fraction schedule, Timmerman constraints were used.^20^

**TABLE 1:** Dose limits for organs at risk at highly hypofractionated radiotherapy.

ORGAN	5 fractions	3 fractions
Small bowel	Dmax^a^< 35 Gy	Dmax < 25.2 Gy/ V 17.7 Gy < 5 cm^3^
Bladder	Dmax < 38 Gy/ V 18.3 Gy < 15 cm^3^	Dmax < 28.2 Gy/ V 16.8 Gy < 15 cm^3^
Rectum	Dmax < 38 Gy/ V 25 Gy < 20 cm^3^	Dmax< 28.2 Gy/ V 24 Gy < 20 cm^3^
Skin	Dmax < 38.5 Gy/ D 10 cm^3^< 36.5 Gy	Dmax < 33 Gy/ D 10 cm^3^< 31 Gy

^a^ Max point dose defined as ≤0.035 cm^3^ ^.^

Based in SABR UK protocol,^16^ Timmerman,^20^ HYTEC^21^ publication, and AAPM report 101.^22^

### 2.3 Statistical analysis

The CK and Halcyon treatment plans were compared as cohorts using a paired Wilcoxon signed rank test of the dosimetric indexes. All statistical routines were scripted in the software R 4.2.1. A *p*‐value of 0.05 was taken as the significance threshold.

Statistical analysis was performed to evaluate the comparative performance of Halcyon and CK treatment plans below (B) and above (A) a given PTV volume to establish an absolute PTV volume threshold that could be used as a predictor of the quality of the Halcyon plan. A receiver operating characteristics (ROC) curve that computed outcomes for different decision thresholds was determined. The dosimetric indexes were categorized for every point according to their relative difference between the paired data points. If the resulting difference was below 10%, the data point was classified as 0 or 1 otherwise. Ten percent threshold value was chosen as a minimal value providing statistically significant separation between tested groups on NCI parameter. Afterward, the specificity and sensitivity for a PTV volume or threshold were computed. This procedure was repeated for all values in the range of PTV volumes, thus creating the ROC curve. The best threshold is chosen from the highest point of sensitivity and specificity. According to Mandrekar,^23^ the area under the curve (AUC) of the ROC graph indicates the accuracy of the test. An AUC result of 0.8–0.9 is considered excellent, a value of 0.7–0.8 is considered acceptable, and below 0.7, the test is taken as poor.

The groups CK‐H (CyberKnife—Halcyon), H‐H, and CK‐CK in both regions of a given PTV volume or threshold (below and above) were compared to complete the statistical evaluation. The *optimal* threshold was defined as the cut‐off point that classifies the pair of groups H_B_ ‐H_A_ and H_B_ ‐CK_B_ as significantly different (*p* < 0.05) and H_A_‐CK_A_ as not significantly different, according to their respective Wilcoxon signed rank test.

### 2.4 Plan comparison

The plans were compared using dosimetric indices such as the new conformity index (NCI), the conformity index (CI), the modified gradient index (MGI), the ratio of the prescribed isodose volume (PIV) to the target volume that receives the prescription dose (TV_PIV_), and the percentage of the target volume covered by the prescription dose (%PIV). These parameters are defined as follows:

(1) $$NCI\;=\frac{TV*PIV}{TV_{PIV}^{2}}\;,$$

(2) $$CI\;=\;\frac{PIV}{TV}$$

and

(3) $$MGI\;=\;\frac{PIV_{50\%}}{TV_{PIV}},$$

where TV is the target volume, and PIV_50%_ represents the isodose volume at half of the prescription dose.

The CI has commonly been reported as the ratio of the prescribed isodose volume to the target volume.^13^ However, it did not consider the possible undertreatment of the target. To solve this vagueness, the Paddick conformity index (PCI) was proposed.^15^, ^24^ This index was later redefined by its reciprocal, known as the NCI parameter, and it is an international standard measure endorsed by ICRU today.^15^

The ideal values for both NCI and CI parameters are below 1.2, with a mandatory value below 1.5 according to institutional guidelines. In Table 2 are summarized requirements for dose spillage or the inverse of the selectivity index (PIV/TV_PIV_) and the MGI parameter.^15^ Furthermore, according to institutional guidelines, coverage (%PIV) should be higher than 95% for SBRT plans.

**TABLE 2:** Prescription dose spillage requirements and modified gradient index requirements for non‐lung sites for PTV volumes < 20 cm.^3^

	Target	Tolerance	Minor deviation
Vol (100%)/ PTV V100%	1.20	<1.25	1.25–1.40
Vol (50%)/ PTV V100%	5.5	7.5	7.5‐9.5

To evaluate the difference in possible acute and chronic complications of the OARs between plans, the normal tissue complication probability (NTCP) was determined for each plan using Lyman Kutcher Burman (LKB) and relative seriality (RS) models.

The LKB model is a recursive algorithm which uses tolerance dose data of normal tissues and calculate the complication probability from the corresponding dose‐volume histograms (DVH). The DVH is reduced to a power‐law dependency called equivalent uniform dose (EUD). This DVH summary measure is the dose that, when delivered homogenously on the whole organ will lead to the same NTCP as the given non‐uniform dose. It contains the exponent *n* which reflects the volume effect of the OAR. The model describes two additional parameters: the tolerance dose to the whole organ which leads to a complication in 50% of the population *TD50*, and the steepness of the sigmoid‐shaped dose response *m*.

The relative seriality model proposes an explicit dependence of the architecture of an organ. The organ is considered as being composed of an *nxm* matrix of parallel and serial subunits whose local response is considered in the overall complication probability. The local response of each subunit is obtained from the probability of no cell surviving derived from an approximation to Poisson statistics. The model includes the parameter s representing the relative seriality of the tissue, being *s* = 1 for a structure in parallel and *s*→0 for an organ considered in series. In addition to this parameter, the model depends on tolerance dose TD50 (same as in LKB model) and the normalized dose response gradient γ.

NTCP calculation was performed for the bladder, small bowel, and rectum, using the parameters of the LKB model^25^ (see Table 5) fitted to the radiation tolerance data of Emami et al.^26^ For the small bowel (17 patients), this estimate applied the parameters reported by Burman et al.^27^ with endpoint obstruction/perforation. For the rectum (six patients), the parameters of Tucker et al.^28^ with grade ≥2 RTOG endpoint were used. For bladder (11 patients), the parameters of Cheung et al.^29^ with the endpoint of any chronic genitourinary (GU) toxicity within 2 years after the treatment were used. An additional NTCP evaluation was performed employing the RS model^30^ and the fitted parameters reported by Gukenberger et al.^31^ (Table 6). The endpoints were severe proctitis/necrosis/fistula, stenosis for the rectum, symptomatic bladder contracture and volume loss for the bladder, and obstruction perforation/ fistula for the small bowel.

An inner layer of body structure of 5 mm thickness was created to assess the skin dose. The maximum dose in this structure as well as the dose in a volume of 10 cm^3^ were evaluated.

No authorization from the regional ethics committee was needed according to the institutional review board response to filled form BASEC‐Nr. Req‐2022‐01393. All patients provided their informed consent to anonymous data use.

## 3 RESULTS

### 3.1 Dose and fractionation

Table 3 indicates the mean and range of the fractionation schema used in the treatment plans for both cohorts of treatment modalities. The prescription isodose values in both groups are shown in Table 3.

**TABLE 3:** Target volume and treatment variables in a sample of 20 patients and 21 plans with pelvic LN lesions.

Quantity	Total dose (Gy)	Num. fractions	%PI^a^ (CK)	%PI (Halcyon)	PTV^b^ vol. (cm^3^)
Mean ± SD	36.5 ± 2.5	3 ± 1	75.1 ± 3.8	70.3 ± 1.8	4.0 ± 2.6
Range	26–40	2, 3, 5	69–80	66–75	1.1‐10.7

^a^PI, Prescription isodose; ^b^PTV, Planning Target Volume.

### 3.2 OARs and target volumes

Six, 11, and 17 patients had the rectum, bladder, and small bowel delineated, respectively. Detailed information on the PTV volumes is consigned in Table 3. Nineteen patients with single tumors and two patients with dual lesions were evaluated.

### 3.3 Dosimetric parameters

**FIGURE 1:**
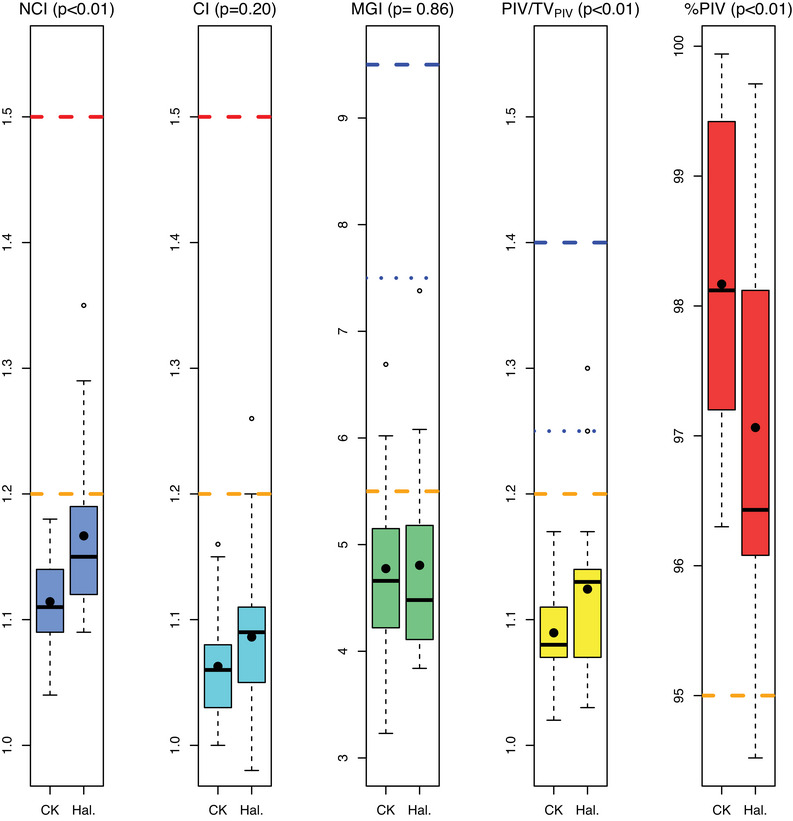
Boxplot comparison of the dosimetric indexes from 21 CK treatment plans against the same replanned treatments with Halcyon (Hal) system. The *p*‐values were obtained from their respective non‐parametric paired Wilcoxon test. The mean values of the dosimetric parameters in each group are shown as black dots. Indexes limits of the ideal values (horizontal dashed orange lines in 1.2 and 95%), mandatory thresholds (horizontal dashed red lines in 1.5), tolerances (horizontal dotted blue lines in 1.25 and 7.5), and minor deviation limits (horizontal dashed blue lines in 9.5 and 1.4) are depicted for each parameter if available.

**FIGURE 2:**
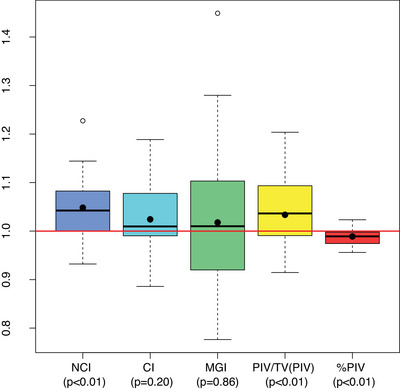
Boxplots of the dosimetric indexes ratio (Halcyon/CK) from CK and Halcyon treatment plans performed on the same set of patients. The mean values of the ratios are represented by black dots.

The dosimetric indices are shown in Figure 1, where the NCI, MGI, PIV/TV_PIV_, and %PIV values obtained from the CK and Halcyon plans are shown. The statistical results are shown in Table 4. A statistically significant difference was found for the parameters NCI, PIV/TV_PIV_, and %PIV (*p* < 0.05). A relative comparison of the indexes (Figure 2) differs in a range of 0–20% for most of the plans. The larger but not statistically significant difference is observed for the MGI parameters and the lower for the %PIV indices.

**TABLE 4:** Evaluation of the dosimetric parameters of CK and Halcyon plans for 20 pelvic SBRT patients.

Parameters	CK	Halcyon
NCI	1.11 ± 0.04	1.17 ± 0.07
CI	1.06 ± 0.05	1.09 ± 0.06
MGI	4.78 ± 0.83	4.81 ± 0.93
PIV/TV_PIV_	1.09 ± 0.04	1.12 ± 0.06
%PIV	98.17 ± 1.31	97.06 ± 1.49

For each parameter, its mean and standard deviation are shown.

Seventy‐one percent (15 plans) of Halcyon plans and 81% (17 plans) of CK plans have met all the ideal criteria. Twenty‐four percent (five plans) of the HAL and 19% (four plans) of the CK plans fall between the ideal and tolerance values. One Halcyon plan does not satisfy the target coverage criteria of 95% (% PIV) by 0.5% (94.5% coverage was achieved).

### 3.4 Dose to OARs

The set of normal tissue constrains reported in Table 1 were fulfilled by the planned doses of both cohort of plans on the OARs. An additional relative evaluation of the risk of toxicity of normal tissue for both groups of treatment plans for OARs was done by the calculation of NTCP of the bladder, small bowel, and rectum, using LKB and RS models with the parameters reported in Tables 5 and 6. For small bowel with endpoint obstruction/perforation, the mean NTCP for 17 patients was 0.001 ± 0.004% for the CK plans and 0.002 ± 0.008% for the Halcyon treatment plans, with a range of (0–0.02)% and (0–0.04)%, respectively. The median estimate of NTCP in both cohorts, for the rectum and bladder, was 0%, and the mean NTCP was less than 10^−4^%. The endpoints used were grade ≥2 RTOG and any chronic genitourinary (GU) toxicity within 2 years after the treatment for rectum and bladder, respectively. NTCP evaluation, using the RS model, resulted in a mean/median for the CK and Halcyon cohorts of 0% for the rectum and bladder with the endpoints severe proctitis/necrosis/fistula, stenosis and symptomatic bladder contracture and volume loss, respectively. For the small bowel with the endpoint obstruction perforation/fistula, a median NTCP of 0% was obtained for the CK (mean; range: 0.3 ± 1%; 0–4.7%) and Halcyon (mean; range: 0.7 ± 2%; 0–5.1%) plans.

**TABLE 5:** Parameters of the LKB model to obtain an estimation of the risk of toxicity for each OARs.

	n	m	TD50 (Gy)	α/β
Rectum	0.08	0.14	78	4.8
Bladder	0.01	0.022	77.6	4
Small bowel	0.15	0.16	55	10

**TABLE 6:** Parameters of the relative seriality model reported by Guckenberger et al.^31^ ^.^

	D50	Gamma	α/β	Seriality
Rectum	80	2.2	3	1.5
Bladder	80	3	3	0.18
Small Bowel	53.6	2.3	3	1.5

The maximum dose to the skin normalize to the prescription dose were statistically different (*p* < 0.01) for CK (mean; range: 25.1 ± 5.6%; 18.1–45.7%) and Halcyon (mean; range: 16.7 ± 6.2%; 10.1–31.4%) groups. The mean dose in 10 cm^3^ of the skin structure contoured was statistically distinguishable (*p* < 0.01) for both cohorts of treatment plans, with a mean of 8.6 ± 2.3% for Halcyon plans (range: 5.3–15.2%) and 11.0 ± 2.3% for CK plans (range: 7.6–15.8%).

### 3.5 PTV volume as a decision threshold

Table 7 shows the typical parameters of the ROC curve for the different dosimetric indexes. The best thresholds and the range of optimal cut‐off points are depicted in Table 7 for all dosimetric indices when found. The NCI indices have a higher AUC percentage with the best threshold (the highest sensitivity and specificity) of 2.4 cm.^3^ For the %PIV index, no ROC curve could be calculated, as no categorization was possible at the 10% relative difference chosen.

**TABLE 7:** Parameters of the dosimetric indexes ROC curves, optimal and best thresholds.

Index	%AUC [CI]^a^	Best (cm^3^)	Optimal (cm^3^)
NCI	80.9 [55.4, 100]	2.4	2.6–3.6 and 4.2–5.4
CI	53.9 [21.2, 86.5]	2.6	2.9‐3.2
MGI	66.4 [42.3, 90.5]	4.0	not found
PIV/TV_PIV_	68.8 [41.7, 95.8]	4.2	2.6–3.6 and 4.0–5.5

^a^ CI: confidence interval (*p* = 0.05).

Figure 4 demonstrates the boxplots of the dosimetric indices comparing both groups in regions above and below the optimal threshold, obtaining a CK_B_ mean of 1.12 ± 0.04 and H_B_ mean of 1.22 ± 0.08 contrasting with the means above the cut‐off point of CK_A_ 1.11 ± 0.04 and H_A_ 1.14 ± 0.04 for the NCI indexes. Figures 5 and 6 illustrates the dose‐volume histogram (DVH) comparison, together with the colorwash dose representation in the transversal and coronal planes for Halcyon and CK plans with the PTV volumes above and below found optimal threshold.

### 3.6 Delivery

The number of MUs was recorded for each treatment plan. The mean value of the total MUs for the CK and Halcyon treatment plans is shown in Table 8. The direct comparison of the MUs in both groups is not meaningful due to the inherent differences in delivery techniques: a large number of small fields in CK plans versus the 2‐arc VMAT plan with Halcyon. The total delivery time (including set up, initial imaging, and ongoing imaging) for the CK plans was obtained from the plan records (see Table 8), ranging between 18 and 65 min. The total beam‐on time for Halcyon plans was estimated by interpolation of the data of 3‐arc Halcyon SBRT prostate plans reported by Altundal et al.^17^ to the respective MUs, obtaining approximately 6.12 min.

**TABLE 8:** Delivery parameters of the treatment plan cohorts.

Characteristic	CyberKnife	Halcyon
Delivery time (min)	42.4 ± 12.1	6.12^a^
MUs	38714 ± 12576	4597 ± 808

^a^ Obtained from extrapolated values by Altundal et al.^17^

## 4 DISCUSSION

In this work, we investigate the performance of autoplanning for Halcyon SBRT. We contrasted the dosimetric and normal tissue parameters of pelvic LN SBRT lesions with Halcyon and CK modalities, with the caveat that each technique presents a very high mechanical distinction. One should also notice the different dose calculation algorithms used in the Eclipse and Precision planning systems. These differences are beyond the scope of this work, and we focused on the feasibility of using Halcyon by planning comparison.

**FIGURE 3:**
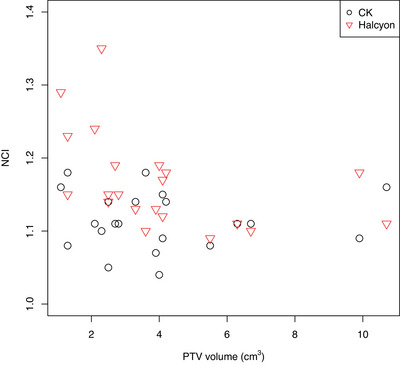
Comparison of the new conformity index (NCI) between CyberKnife (CK) and Halcyon treatment plans for the range of PTV volumes.

**FIGURE 4:**
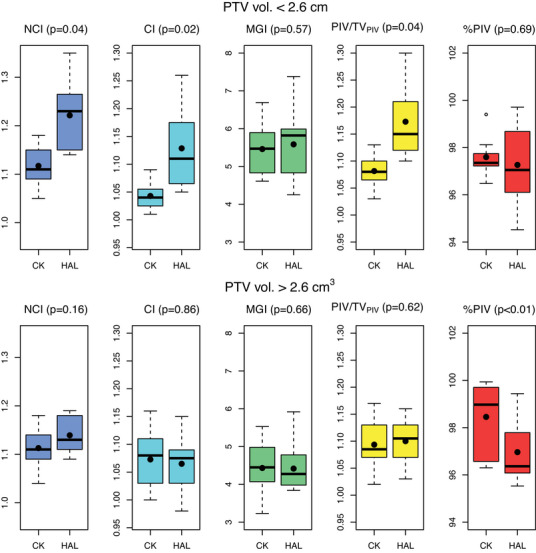
Boxplots comparing the dosimetric indexes from CK and Halcyon treatment modalities below and above the found optimal threshold of 2.6 cm^3^ PTV volume. The mean values of the NCI index are shown as black dots.

Most of the Halcyon (71%, *n* = 15) and CK (81%, *n* = 17) plans met all ideal institutional criteria, with 95% (*n* = 20) of Halcyon and 100% (*n* = 21) of the CK plans being within tolerance. Therefore, the majority of Halcyon treatment plans in our investigation preserved a complete adherence to clinical requirements and became dosimetrically indistinguishable in relation to CK plans above a PTV volume of 2.6 cm,^3^ with the exception of the coverage (Figure 4). These results contrast with the results of Paik et al.,^32^ who reported significantly higher conformity of the non‐isocentric CK treatment plans compared to isocentric VMAT treatments on C‐arm Varian Truebeam Linac, for lesions below 100 cm.^3^ ^32^ Furthermore, the conformity performance of the VMAT/RA plans accomplished in this study surpassed the plan conformity reported by Paik et al.^32^ for the same range of PTV volumes (NCI and CI in Figures 3 and 4). Paik reported 1.13 ± 0.1 (conformity index) and we obtained 1.09 ± 0.06.

Throughout the complete range of PTV volumes, there was statistically significant, but with the plan parameters being within institutional criteria, the non‐clinically relevant difference in NCI, PIV/TV_PIV_, and %PIV and a not statistically significant difference in CI and MGI of VMAT Halcyon plans compared to CyberKnife (CK) plans (Table 4, Figure 1).

Although 2.6 cm^3^ is not the best cut‐off point for NCI (Table 7), it is very close in selectivity and specificity to 2.4 cm^3^ (best threshold) and is part of the set of *optimal* thresholds. According to the AUC results, the NCI index has an acceptable accuracy as a statistical predictor in contrast to the poor accuracy observed in the remaining dosimetric parameters. Based on the AUC results, the *optimal* thresholds, and the best cut‐off point values of all indexes, we conclude that a threshold of 2.6 cm^3^ PTV volume is a good predictor of the dosimetric difference between the CK and Halcyon plans, based on dose conformity (NCI index).

The effect of the treatment plans on the OARs is minimal for both treatment techniques. NTCP calculated with the LKB model for the two cohorts was less than 0.04% for the small bowel, and a mean of 0% was found for the bladder and rectum. In other studies, patients with iliac lymph node metastases were treated with SBRT and the CK system. Higher toxicity rates were obtained for milder endpoints, such as nausea grade 1−2 (18.2%), diarrhea grade 1−2 (4.5%), fatigue grade 1−2 (22.7%), and leucopenia grade 1−2 (4.5%), among others, and no severe toxicities were reported.^14^ Furthermore, the NTCP evaluation performed with the RS model demonstrated the same results, relative to the previous model, for the bladder and rectum within the two groups, but a high risk of toxicity was obtained for the small bowel (≤5.1%) with both CK and Halcyon plans. However, so far no clinical manifestations of such small bowel toxicity have been observed in treated patients. In addition, Halcyon plans showed a better sparing of the skin than CK plans in agreement with the reported outcome in prostate treatments by Altundal et al.^17^

**FIGURE 5:**
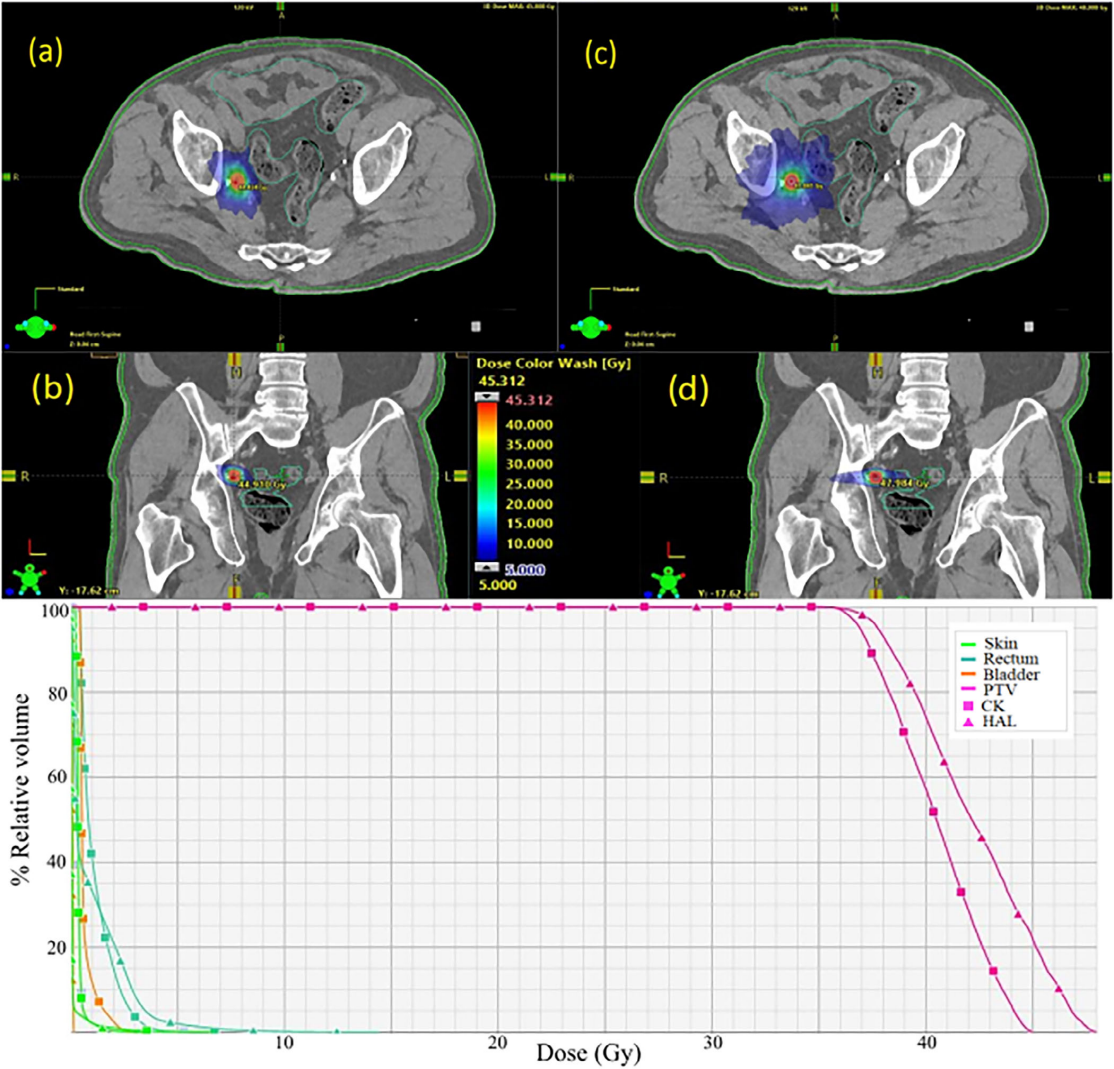
Example of treatment plan for a target volume of 1.1 cm^3^ (below 2.6 cm^3^ threshold). In (a) and (c) are depicted a transversal slice of the dose in CK and Halcyon plans, respectively. In (c) and (d) the dose in coronal plane is shown for the same treatment plans. Below: dose‐volume histogram of CK and Halcyon plans.

**FIGURE 6:**
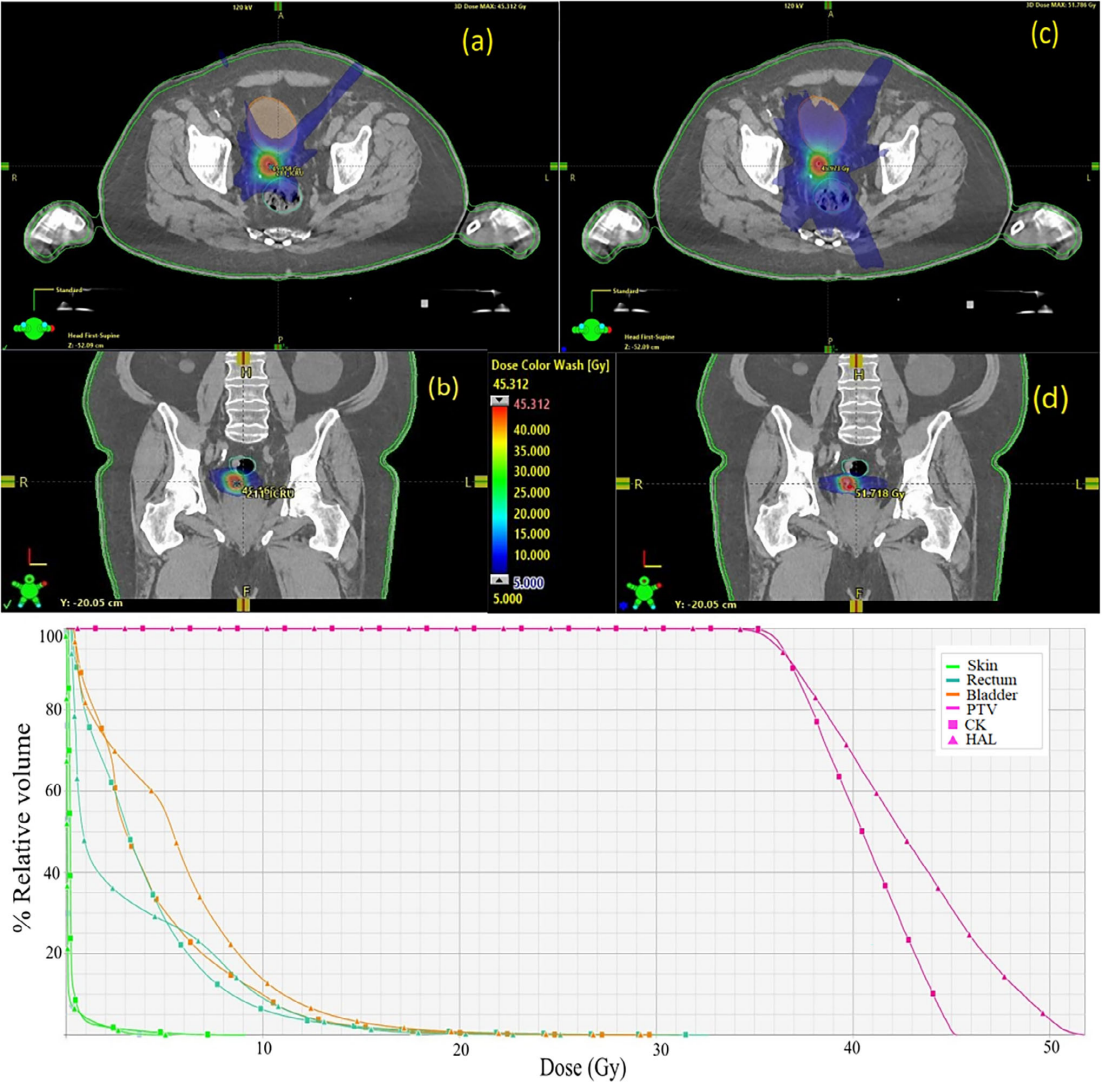
Example of treatment plan for a target volume of 5.5 cm^3^ (above 2.6 cm^3^ threshold). In (a) and (c) are depicted a transversal slice of the dose in CK and Halcyon plans, respectively. In (c) and (d), the dose in coronal plane is shown for the same treatment plans. Below: dose‐volume histogram of CK and Halcyon plans.

The DVH comparison (Figures 5 and 6) demonstrate that there are no major differences in the dose to OARs in CK and Halcyon plans both above and below optimal PTV volume threshold, however the doses to the targets are distinguishable for both plans. This outcome is due to, on one hand, the difference in the dose prescription isodose and on the other hand, to differences in conformity between treatment plan groups.

A significant disadvantage of CK over Halcyon treatments is the long delivery time (Table 8). Long delivery times can cause patient discomfort and possibly compromise the treatment accuracy due to internal anatomical changes (for example, caused by bowel gas motion).

One of the limitations of this study is its small sample size (20 patients and 21 treatment plans), which could limit the applicability of these results to larger cohorts of patients. An analysis of treatment plans focused solely on dosimetric comparison inevitably disregards the scope of the effects of mechanical differences in the treatment modalities. These include the evaluation of the effect of the differences between MLC and cone collimators, the 6 degrees of freedom CK plans versus the coplanar Halcyon plans, and the number of fields of each technique on the precision of the treatment plan delivery.

## 5 CONCLUSION

This work demonstrated the feasibility of the ring‐mounted Halcyon Linac treatment plans for pelvic LN SBRT. Halcyon plans met clinical requirements and had much shorter delivery times. Although CyberKnife plans showed higher coverage and conformity, these differences were not clinically significant, meaning that both set of plans satisfy the plan criteria of SABR UK protocol and ICRU 91 report. The results of this study indicate that the Halcyon treatment plans are dosimetrically comparable to the CK plans for PTV volumes above the found *optimal* threshold of 2.6 cm,^3^ while below this cut‐off point a better dosimetric performance of CK is observed. This new parameter could be used as an essential decision criterion distinguishing treatment modality that best suits the target's characteristics.

## AUTHOR CONTRIBUTIONS

All authors have contributed to this work.

## CONFLICT OF INTEREST STATEMENT

The author have no other relevant conflict of interest to disclose.
